# Living with pelvic organ prolapse: voices of women from Amhara region, Ethiopia

**DOI:** 10.1007/s00192-016-3077-6

**Published:** 2016-07-30

**Authors:** Janne Lillelid Gjerde, Guri Rortveit, Mulu Muleta, Mulat Adefris, Astrid Blystad

**Affiliations:** 1 Department of Global Public Health and Primary Care, University of Bergen, P.O. Box 7804, 5020 Bergen, Norway; 2 Department of Obstetrics and Gynecology, Haukeland University Hospital, Bergen, Norway; 3 Research Group for General Practice, Department of Global Public Health and Primary Care, University of Bergen, Bergen, Norway; 4 Research Unit for General Practice, Uni Research Health, Bergen, Norway; 5 Women and Health Alliance (WAHA) International/Gondar Fistula Center, Gondar University Hospital, Gondar, Ethiopia; 6 Department of Obstetrics and Gynecology, School of Medicine, University of Gondar, Gondar, Ethiopia; 7 Centre for International Health, Department of Global Public Health and Primary Care, University of Bergen, Bergen, Norway

**Keywords:** Ethiopia, Experience, Low-income setting, Pelvic organ prolapse

## Abstract

**Introduction:**

The objective of the study was to explore how women with symptomatic pelvic organ prolapse in a low-income setting explain, experience, and handle the potential practical and social consequences of the condition.

**Methods:**

An explorative qualitative design was employed using in-depth interviews in the data collection. A total of 24 women with different degrees of symptomatic pelvic organ prolapse were included; 18 were recruited at the hospital and 6 from the community. Fieldwork was carried out in the Amhara region of northwest Ethiopia in 2011 and 2015.

**Results:**

The informants held that the pelvic organ prolapse was caused by physical strain on their body, such as childbirth, food scarcity or hard physical work, particularly during pregnancy and shortly after delivery. Severe difficulties and pain while carrying out daily chores were common among the women. The informants used a variety of strategies to manage their work while striving to avoid disclosure of their condition. Disclosure was related to embarrassment and fear of discrimination from people living close to them, including the fear of being expelled from the household. Most of the informants, however, experienced substantial support from relatives, friends, and at times also from their husband, after disclosing their condition.

**Conclusions:**

The study highlights how symptomatic pelvic organ prolapse may severely affect women’s lives in a low-income setting. The condition is perceived to be both caused by and aggravated by the heavy physical burdens of daily work.

## Introduction

Existing knowledge on the prevalence, risk factors, and consequences of living with prolapse in low-income settings is scanty. The fertility rate is higher, and the access to obstetric care is far more restricted than in more affluent settings, factors that may have implications for the risk of pelvic floor disorders [1]. It has been argued that prolapse may be more common in resource-constrained settings owing to risks related to these established factors and to heavy physical burdens, and that the condition may affect daily life more severely than in high-income settings [2]. The few available publications on prolapse in low-income settings illustrate how the suffering related to conditions of prolapse severely affects the women’s emotional well-being and the relationship with their husbands, not the least because of the inability to perform chores expected of them [3–6]. Depressive symptoms among women with prolapse have also been reported from northwest Ethiopia [7].

The current study was conducted in the Amhara region of northwest Ethiopia, where the median female age at first marriage is 15.1 years [8]. The major ethnic group in the area is the Amhara, who practice Ethiopian Orthodox Christianity and speak Amharic as their first language [9]. Skilled personnel attend only 10 % of all births in rural areas; the fertility rate is 4.1 children per woman [10], and the maternal mortality ratio is 497 deaths per 100,000 live births in Ethiopia [11]. Health facilities in the rural areas are poorly equipped, with inadequate emergency obstetric services [12]. Additionally, only 40 % of the women in the region are literate [10].

With these indicators, Ethiopia is a low-income setting that may be well suited to the study of women’s experiences and the management of prolapse. The present study aimed to explore how women living with prolapse in northwest Ethiopia explain, experience, and handle the potential practical and social implications of prolapse.

## Materials and methods

The first part of the study, carried out in 2011, included 8 women with prolapse, 6 of whom were recruited from the sample of women identified with prolapse through the Dabat Incontinence and Prolapse (DABINCOP) pilot study [13, 14]. The second part of recruitment took place in 2015 at Gondar University Hospital, where all 16 informants were admitted and underwent surgical treatment for their condition. The informants were invited to participate in the study based on their home district, age, and length of time suffering from the prolapse, aiming to obtain a wide variety of experiences and the possibility of following them up at a later stage.

Both sessions of fieldwork were carried out by the first author. The design during both study periods employed a qualitative explorative approach with in-depth interview as the main data collection approach. Semi-structured interview guides with open-ended questions mainly concerning experiences of the potential practical or social consequences of living with prolapse were employed during the data collection. Both research assistants were from the area and were familiar with language, culture, and respectful conduct in the area. All the interviews were conducted in Amharic with continuous translation to English. The interviews lasted 1–2 h and were aimed at letting the informant speak freely and without interruptions. The six interviews carried out in the community took place in the informants’ homes and outside the health centre or health post located in their village. The remaining informants were all interviewed in a private room at the hospital ward where they were admitted. A digital recorder was used to record the interviews, and all interviews were transcribed verbatim, with continuous checks by the research assistants to ensure that the content was correctly captured and culturally specific expressions retained. The interviews were transcribed in Amharic, followed by translation into English.

The analysis process was based on the principles of systematic text condensation [15]. The analysis took place throughout the data collection phase and during a rigorous analysis phase after completion of the fieldwork. The data material from 2011 was organized and coded manually, whereas the data material from 2015 was organized and coded by the use of NVIVO, a qualitative data analysis computer software package.

Ethical approval for both study periods was obtained from the Regional Ethics Review Board in Western Norway and from the Institutional Ethical Review Board at the University of Gondar, Ethiopia. The aim and purpose of the study, and the contents of the consent form, were read aloud to all informants before the interview. Written or oral consent to participate was obtained depending on literacy status. Three women who were asked to participate declined shortly after being approached. None of the participants withdrew their consent.

## Results

A total of 24 women with symptomatic prolapse, ranging from stage II to IV, were included in the study. All of the informants were from the Amhara region and nearly all were Orthodox Christians. The median age of the informants was 40.25 years (range 24–65). 18 informants were married, whereas the remaining 6 included two widows and four divorcées. The mean age at first marriage and first delivery was 13 years (range 7–30) and 19.2 years (range 13–32) respectively. The majority (22) of the informants were multiparous, with the mean number of deliveries being 4.4. Furthermore, 20 of the informants had delivered all of their children at home. Twenty of the 24 informants had never gone to school and were illiterate, and 21 were unemployed and mainly carrying out housework and helping out with farming activities. The 24 women with prolapse had stage II (2 informants), III (20 informants) or IV (2 informants) according to the simplified pelvic organ prolapse quantification staging system (S-POP) [14, 16]. Approximately half of the women had had prolapse for the last 10 years or more, including 7 who had lived with the condition for more than 20 years. Eleven of the women reported difficulties urinating because of the prolapse, whereas five suffered from urinary incontinence, ranging from mild to severe leakage.

### Conceptualizing the condition

Most of the informants argued that physical strain on their body, such as childbirth, labor or food scarcity, had caused the prolapse. One informant stated: “Most of the time it happens due to delivery, because most of us didn’t go to a health facility for delivery. We deliver at home where we go through labor for two to three days. When we finally give birth it’s so hard that the uterus will ‘go out’” (39 years, prolapse stage II). Also, early age and multiple pregnancies were mentioned as causes. Others related the prolapse to their heavy workloads: “I am busy working and carrying heavy goods. Due to this my uterus comes out” (47 years, prolapse stage IV). “Our area is mountainous, and I used to help him [husband] dig while he was holding the ox ploughing. I realize that I have been exposed to this heavy workload, and I keep thinking that if I had refrained from working that much, I could have been fine” (40 years, prolapse stage III). Some, moreover, mentioned the lack of appropriate nutrition as putting the body under continuous strain, causing the prolapse: “It is because of scarcity of food. Like many other mothers I don’t get proper food” (39 years, prolapse stage III). A few informants blamed their condition on God’s will or linked it to the agency of the spirits. One informant said: “I considered it as God’s anger. What else shall I think?” (50 years, prolapse stage III). Another explained her experiences of “bad eyes”: “People’s eyes can make you disabled or can make you die. My sister-in law...Her eyes are bad...It is because of her that I’m sick” (33 years, prolapse stage III).

### Challenges of daily life

In particular, the married women in the study were responsible for all household chores, including childcare and cooking, and the fetching of water and firewood. Fetching water often involves walking several hours a day holding 20–30 L of water on one’s back. This is considered women’s work, and is an integral part of everyday life for women and girls in this area. Married women typically also help their husband in farming activities until the children are old enough to help out. Almost all of the informants emphasized the constant burden of work and the challenges this caused owing to their condition. “I do weeding, grinding, harvesting, the fetching of water and collection of firewood. A woman doesn’t get rest” (45 years, prolapse stage III). Another woman explained: “My uterus comes out while I am working or when I am walking. We don’t even get rest during pregnancy or right after delivery” (39 years, prolapse stage III). While working, the prolapse was often experienced as very uncomfortable, and sometimes painful, as expressed by a large number of informants: “It is especially severe when I walk for long. I do manage to control the problem, although with pain” (40 years, prolapse stage III). “When I walk, it [the prolapse] rubs against my thighs and the skin peels off” (65 years, prolapse stage III). For a few of the women the challenge was experienced as worst in relation to urination: “Whenever the uterus comes out and I go to urinate, it [the urination] is blocked. This year was terrible; it [the prolapse] blocked me; pressed me from inside. It was like giving birth” (33 years, prolapse stage III). Some of the women talked about how they no longer were able to fulfill the social role expected of a wife: “I used to invite people to come to our home on St. Mary’s day, but then I stopped because I was unable to grind, to serve food and to host guests” (42 years, prolapse stage III).

### Sexual implications

Many of the married informants continued being sexually active as long as they managed, although commonly with pain: “I used to push my uterus inside and do the intercourse. Even though I feel sick, what can I do? It is marriage” (33 years, prolapse stage III). At times however, the prolapse prevented women from having sexual intercourse: “When my uterus is out, I usually sleep alone. When I’m in that situation I tell him that I’m ‘on period’ or that I’m sick” (35 years, prolapse stage III). Other women had not been able to have sex for years: “When it [the prolapse] started to become sore I refused him. It has been five or six years since I started to forbid him to have sex with me. He didn’t say anything; he started looking for another woman” (45 years, prolapse stage III). Another informant got support from her husband: “When I told him I have been suffering a lot because of it [the prolapse] he didn’t push me to have sex with him anymore. He understood the problem, and we stayed one year without intercourse” (33 years, prolapse stage III). All four divorcées explained that they got divorced due to their prolapsed condition. One of them explained: “Sometimes when we were about to have sex he saw that my uterus was out and we had to stop. When that happened repeatedly he started to feel discomfort. He then gathered my family and relatives and gave me money. We got separated without having any conflict or discussion” (39 years, prolapse stage II).

### Managing the condition

Despite the challenges, all the women interviewed strove to continue with their lives and not the least with their daily chores: “Since I have a husband and children I have to work, so I grind and I prepare the meals even if I feel the pain” (42 years, prolapse stage III). “Even though I don’t work as I used to, I still work as much as I can” (50 years, prolapse grade III). Several practical strategies for managing their condition were employed. One woman explained: “When I walk I feel like something is pushing me down and I get a cramp at my waist. When that happens I sit down and take a rest until the prolapse goes back inside. Then I can continue walking. This is how I live” (45 years, prolapse stage III). Another woman explained: “Whenever I can, I try to work while sitting down” (40 years, prolapse stage III). However, this was not the best solution for all: “It is difficult to sit so I usually sit on folded clothes” (42 years, prolapse stage III). Yet another woman explained how she handled difficulties urinating: “Since it [the prolapse] blocks my urine, I push it in with my hand in order to urinate” (33 years, prolapse stage III). For some, however, pushing the prolapse back didn’t help for long, and at times, not at all. A woman with the most severe stage of prolapse explained: “I moisten it with oil. When I use the oil, it becomes soft and I can sit, and it will draw back a little. After that I can walk again, but it is still difficult to make long walks” (47 years, prolapse stage IV). Other practical strategies included delegating work-related chores to children, or at times finding excuses for not doing particular chores.

### Difficulties of disclosure

Disclosing the condition was experienced as challenging for nearly all of the women. Suffering from prolapse was regarded as extremely shameful, and disclosing the condition was believed to potentially have severe implications. One informant at the hospital, after having discussed the issue among other women with prolapse explained: “In my home town everyone is equal and healthy. Talking about our problems will make people ignore us. So we are scared to be open about our health problems” (45 years, prolapse stage III). Most had kept it to themselves for years before deciding to disclose: “I used to think that it was disgusting to talk about and that no one else in my hometown had a similar problem” (65 years, prolapse stage III). A few informants had in fact never disclosed their condition to anyone close to them: “I asked myself what the use was of telling other people if there is no solution. I was scared and wondered what was happening to me. Since I didn’t know what it was I just kept quiet” (40 years, prolapse stage III). Most feared the social consequences: “If they hear about it they would discriminate me and talk about me behind my back” (40 years, prolapse stage III). Despite this fear of discrimination or rejection from people close to them, more than half had chosen to disclose to trusted family members, such as sisters, mothers or a child: “I have told only my mother and sister. They sympathized with me when I told them” (40 years, prolapse stage III). “My daughter insisted that I tell her about my problem. I told her that it was no use telling her because there is nothing we can do anyway. She told me that at least she could share my worry and pain” (47 years, prolapse stage IV). Some informants had told a friend or a neighbor: “My neighbor told me a year ago that she has prolapse, so then I decided to tell her that I have the same condition. Both of us were thinking that we were the only ones having this problem. Then we openly talked about our secret which we had kept for so long. We felt so sorry for one another” (40 years, prolapse stage III).

Of the 18 married women, more than half had disclosed to their husbands. Most of those chose to do so at a point when the condition had become serious and very difficult to hide: “I told him recently when it got worse. I told him that I was caught by a disease that people don’t know about. He got angry and asked me why I had hidden it for such a long time and didn’t tell him before” (35 years, prolapse stage III). However, there were women who regarded it as unthinkable to disclose to their husbands. One explained: “My husband is not concerned about my health. I know of many women who were victims of gossip, and I know some who were chased away from their home due to this kind of problem. I am very concerned that one day he might know or suspect that I have this problem” (39 years, prolapse stage III). One of the divorcées explained her previous husband’s reaction when disclosing to him: “He insulted me. He said I was less than a person” (40 years, prolapse stage III).

## Discussion

Most of the women explained that their prolapse was linked to delivery. Additionally, the informants indicated that demanding chores were carried out during pregnancy and shortly after delivery, causing undue strain on their bodies. In a study from rural Nepal, women similarly reported that they believed that childbirth, lack of rest during the postnatal period, and heavy lifting when carrying out daily house chores were the main reasons for their prolapse [4]. Findings from research in India similarly revealed that hard work soon after delivery was common, and was an important factor associated with uterine prolapse among the informants [5]. While childbirth is a well-known risk factor for prolapse [17, 18], little is documented regarding hard physical labor as a risk factor from studies in high-income settings, most likely because physically demanding labor simply is less common in these settings. In the DABINCOP pilot study carried out in the current research area it was found that carrying heavy objects for 5 h or more daily was associated with anatomical prolapse [14]. The highly demanding physical labor that women perform in many resource-constrained settings suggests work load as a factor of particular relevance to be explored in population-based studies in similar contexts.

Despite the severe difficulties and pain experienced when carrying out expected chores, the women in the study strove to manage the tasks while simultaneously working hard to hide their condition. Although all the study participants feared the consequences of a general disclosure of their condition, and some had very negative experiences following disclosure, most of the women who had disclosed encountered great support, commonly from a mother or sister, but at times also from the husband. Study findings from research in Uganda report that half of the women with prolapse did not receive any support from their husbands, and several got divorced as a result of the disclosure [3]. Similarly, in a study from Nepal, some women with prolapse experienced humiliation and harassment both from their husbands and from other family members [4]. In rural Ethiopia, women’s power to make decisions is known to be hampered by illiteracy and early marriage [8]. The immense shame and perceived stigma connected with prolapse and with other pelvic floor disorders in this area [7, 13, 14] is likely to be at least partly related to the lack of health education available to women in rural and sub-urban areas. This makes the symptoms of prolapse unfamiliar, and in turn nourishes the dynamics of secrecy. The DABINCOP pilot study suspected underreporting by the informants owing to substantial discrepancy between the reported and the clinically observed prolapse [14]. This is likely to be at least partly related to the perceived shame and stigma leading to secrecy among women with prolapse.

Kleinman [19] reveals how pain and suffering are rarely limited to individual sufferers, and commonly extend to one’s family and to the broader social network. In the case of prolapse, the present study suggests that the entire family might be affected if a woman is unable to provide the household with water, firewood or food; children may miss school if they have to take on their mothers’ work; and it would affect the children severely if their mothers were expelled from the household. This scenario indicates that a potential health information program for prolapse may need to involve not only the women themselves. Husbands, as the prime decision-makers, commonly have substantial influence over how women in practice can handle the condition. Such concerns, however, need to be carefully balanced against the potential implications of disclosure for the particular woman.

Living in a setting with high illiteracy rates—where women have little knowledge about common maternal morbidities and have limited decision-making power, where household chores include heavy manual labor, and where health system resources to meet even minor health challenge are severely limited—living with a prolapse has very severe implications. In addition to the need for further research on the prevalence and risk factors of prolapse in resource-constrained settings, there is also need for increased knowledge on what would enable women to reach the public health care system for treatment of the prolapse. Such knowledge may importantly inform the development of effective future interventions for this prevalent and readily treatable maternal health challenge in Ethiopia and in other severely resource-constrained settings.

An important limitation of the present study is the extreme taboo this topic represents in this study setting. Owing to the shyness of many of the informants, we may have lost some of the depth of the illness narration. Despite several longer research-based stays in Ethiopia, the first author has limitations in terms of socio-cultural and language competence. As commonly pointed out, there is, however, also a possibility that the informants may have been more open about their condition than they would have been in encounters with local researchers, as a stranger is presumably not familiar with the culturally embedded taboos and stigma surrounding such conditions.
